# The Boost Team: transforming outreach for female veterans in rural communities

**DOI:** 10.3389/frhs.2025.1617105

**Published:** 2025-07-29

**Authors:** Jenny Krause Cohen, Kara Zamora-Rogoski, Caitlin McLean, Mariam Jacob, Tara Stacker, Caroline Dancu, Jennifer Childers

**Affiliations:** ^1^ United States Department of Veterans Affairs, San Francisco VA Health Care System, Veterans Health Administration, San Francisco, CA, United States; ^2^ Department of Medicine and Department of Clinical Informatics and Digital Transformation, University of California, San Francisco, CA, United States; ^3^ United States Department of Veterans Affairs, VA Central Western Massachusetts Healthcare System, Veterans Health Administration, Leeds, AL, United States; ^4^ Veterans Integrated Service Network 21, Pleasanton, CA, United States

**Keywords:** mixed methods, rural veterans, women veterans, outreach, primary care redesign

## Abstract

**Introduction:**

Prior studies show that rurality and sex act synergistically resulting in poor health outcomes for rural female Veterans in large part due to challenges utilizing timely and convenient health care services. We conducted a mixed-methods quality improvement evaluation of a novel outreach program within the Veterans Health Administration (VHA) that provides clinician-driven outreach to women Veterans served by rural VHA clinics. Our goal was to understand the impact the program has on rural female Veterans' access to and engagement in VHA care as well as the impact the program has on health system leaders and rural VHA clinic staff.

**Materials and methods:**

In 2022, we developed a clinician-driven outreach program known as The Boost Team. The Boost nurse practitioner (NP) “cold called” female Veterans within a regional VA health care system and provided real-time clinical care and linkage to VHA services. Outreach calls were completed with 543 Veterans and metrics on call attempts and services rendered were tracked. Of Veterans who received the outreach, 58 completed a telephone survey using the Patient Empowerment Engagement Activation Survey (PEEAS), and 21 completed a semi-structured interview. Interviews focused on experiences with the NP, Boost's impact on access to and experience of VHA care, and suggestions for program improvement. Health system leaders (HSLs; *n* = 11) and rural VHA clinic staff (*n* = 5) completed interviews focused on barriers and facilitators to wider implementation of the Boost Program.

**Findings:**

The most common needs addressed during outreach calls included new referrals to specialty care, completion of outstanding health care maintenance (e.g., age-appropriate cancer screening), coordination with primary and specialty care, assistance with medication management, and reviewing diagnostics (e.g., labs, imaging). PEEAS data demonstrated strong agreement across all categories (median = 5 or “strongly agree”). Veterans, HSLs, and clinic staff reported that Boost outreach increased trust in VHA among Veterans and provided necessary support for under-staffed clinics.

**Conclusions:**

Clinician-driven outreach is a powerful tool to improve access to and engagement in care for rural female Veterans and provides necessary support to rural VHA clinics. Future efforts include expanding geographic range of Boost and better characterizing populations of veterans that most benefit from clinician-driven outreach.

## Introduction

Rurality is a known driver of health inequity among Veterans (1, 2) due to challenges accessing and coordinating care (3, 4). Rural Veterans are at higher risk for avoidable hospitalizations, suffer from lack of equitable Veterans Affairs (VA) disability claims adjudication, and experience more delays in care, less access to clinical pharmacy support, higher rates of disease-specific morbidity and mortality, and higher rates of unmet mental health needs as compared to urban peers (1, 2). Data also show that rurality and sex can work together to lead to poor health outcomes. Health gaps seen in rural Veterans and in female Veterans are linked to poor access to Veteran Health Administration (VHA) services, including lack of primary and mental health care (1, 2, 5, 6). Furthermore, female Veterans are less likely to use VHA services, including primary care, compared to male peers (5, 6). Primary care is the foundation for a functional health system and is necessary to ensure positive health outcomes and comprehensive healthcare (7, 8). Combating poor access and under-utilization of VHA primary care among rural female Veterans is necessary to improve wellness and health outcomes.

While healthcare challenges continue to negatively impact rural Veterans, the post-COVID era of telehealth has ushered in a new cadre of novel centralized care programs that are effective in the VHA setting and allow healthcare providers to provide distributive services to Veteran populations over wide swaths of geography. A 2019 systematic review showed that patient navigation services and telephone calls administered by lay healthcare extenders/non-clinicians increase colorectal, breast, and cervical cancer screening among populations facing challenges accessing care (9). Similarly studies from the maternal health and women's health ecosystems showed that care coordination conducted by lay navigators, community health workers, and registered nurse all lead to improved mental and physical health outcomes, patient satisfaction, utilization of care, and completion of specialty care and diagnostics (10). Literature from the mental health and primary care fields reported that telephonic clinician-driven outreach can result in more robust and meaningful care engagement than outreach letters or standard in-person office visits (2, 11). Linking Veterans who have historically had poor health outcomes, notably rural female Veterans, to VHA primary care presents a powerful strategy to improve health. Prior work by this team showed that rural female Veterans, rural VHA clinic staff, and VHA health system leaders (HSLs) all identified opportunities to improve access to specialty services (e.g., gynecologic care), and identified a need to educate rural clinic staff and Veterans on VHA services specifically for female Veterans (12).

To address the need to improve access to VHA services specifically for rural female Veterans, we created a telehealth-based intervention focused on clinician-driven outreach. To achieve this goal, one VHA health care system created a novel multidisciplinary team, the Boost Team, including a medical director, nurse practitioner (NP), peer support specialist, and a program coordinator. The Boost NP “cold calls” rural female Veterans enrolled in VHA and provides real-time clinical care to address unmet health needs and linkage back to VHA services. Our feasibility study demonstrated that the Boost outreach calls were acceptable and that rural Veterans, clinic staff, and HSLs found the intervention as a useful strategy to connect women Veterans with VHA care (12).

We conducted a mixed-methods quality-improvement (QI) evaluation of the Boost Team to understand the impact that clinician-driven outreach has on Veteran engagement and activation in care, utilization of VHA services, and general attitudes towards VHA. This manuscript will review the Boost Team's impact and key operational partners' perspectives (HSLs, rural clinic staff, and Veterans) on the Boost NP's role in providing care and helping Veterans navigate barriers to accessing care, providing support for under-staffed VHA clinics, and the intervention's impact on female Veterans' comfort and/or trust in accessing VHA care.

## Materials and methods

### Intervention

Using data from the VHA Corporate Data Warehouse, the Boost Team created an outreach dashboard identifying non-male Veterans enrolled in VHA, assigned to the health system, and whose address geolocated closest to the partnering rural VHA community-based outpatient clinic (CBOC). The dashboard also identified gaps in preventative care such as the need for breast or cervical cancer screening. The Boost NP used the dashboard to call Veterans and provided urgent and primary care. The Boost NP used motivational interviewing to elicit the Veterans' individualized health and wellness goals and provided education, support, and linkage to appropriate resources. The Boost NP aimed to repatriate Veterans to their local VHA CBOC and coordinated follow-up with VHA primary care, or, to honor Veterans' choices and preferences, identified a different VHA clinic or clinician to transition care, if appropriate. The Boost NP worked with local CBOC primary care teams to assist in follow-up and ensure care plans initiated during the outreach call were sustained.

Clinician-driven outreach calls were piloted across five VA primary care catchment areas within one VA health care system from September 2022 through December 2024. Three out of five partnering clinics were in rural areas as designated by urban communing areas (RUCA) and Federal Office of Rural Health Policy (FORHP) definitions, and five out of five clinics serve Veterans residing in rural counties based on RUCA and FORHP definitions (13–15).

Calls were documented in the electronic health record (EHR), workload credits allocated, and Veterans did not have a co-pay for the phone encounter. Programmatic evaluation of the Boost Team's clinical work was seated within broader QI initiatives aimed at improving rural female Veterans' health and determined exempt from IRB review. All work is compliant with VA Handbook 1,200.05 (16). We used a mixed-methods approach to evaluate the Boost Team that consisted of chart review, telephone surveys, and semi-structured interviews with Veterans, rural CBOC clinic staff, and health system leaders.

#### Participants

Non-male Veterans enrolled in VHA and assigned to the health care system were eligible to receive the Boost clinician-driven telephonic outreach intervention. Veterans who received a Boost outreach call were invited to complete a telephone survey and a 30-min semi-structured interview. The Boost study coordinator produced a list of Veterans who were reached by the Boost NP's outreach call between October 2023 and June 2024 and the two qualitative research team members called these Veterans to invite them to participate in a telephone interview. Given that the patient population at VA is predominantly older, we over-sampled for younger female Veterans (under 50 years of age) (17).

### Data collection and measures

Data collection included three components: (1) administrative data; (2) PEEAS survey; and (3) semi-structured interviews.

#### Administrative data

Data including number of call attempts, call conversion, and clinical actions taken during outreach calls were tracked for all Boost calls between December 1, 2022 and December 31, 2024.

#### PEEAS

All Veterans who successfully received Boost outreach between June 1, 2023 and November 1, 2024 were called and invited to complete the Patient Empowerment, Engagement, Activation Survey (PEEAS) (18, 19) two to four weeks after their Boost call. The PEEAS consists of 21 items that are rated on a 5-point Likert-types scale (1 = Neither agree or disagree to 5 = Strongly agree). The PEEAS is the only valid psychometric test instrument to measure a patients' perception of quality of care. Unlike traditional patient satisfaction surveys, PEEAS uncovers nuanced data relating to patients' healthy behaviors, utilization of health care, ability to self-manage chronic conditions, and resilience in the context of health challenges (19). While the survey was initially designed to be used in the hospitalized setting, the tool has been adapted by omitting questions specific to hospital discharge for use in the primary care and outpatient setting (20–25). Similar to prior published work using PEEAS in the ambulatory setting, we removed two questions about hospital discharge in order to use the survey an outpatient context (20–25).

#### Qualitative interviews

We developed original semi-structured interview guides tailored for each key partner group: Veterans, health system leaders and clinic staff members. Two qualitative researchers (KZ, CM) conducted each 30-min interview via telephone or VA Microsoft Teams. Interviews were conducted June 1, 2023 through June 1, 2024 and audio recorded for analysis.

Veterans who participated in a Boost NP outreach call were invited to participate in a qualitative telephone interview within one year of receiving the initial outreach call. Topics covered during Veteran interviews included participants' experience with the Boost NP, the impact of the program on their access to care and to their experience of their care at VHA, experiences seeking sex-specific care at VHA and in the community, as well as suggestions for expanding and improving the Boost program.

Health system leaders and CBOC clinicians and staff were identified through snowball sampling techniques (20) and invited by email to participate in 30-min semi-structured interviews. Topics covered during health system leader and clinic staff interviews included participants' views on program need and impact on patient engagement in care, the appropriateness and acceptability of the intervention, in addition to barriers and facilitators to wider implementation and long-term sustainability of the program.

### Analysis

#### Administrative data

Call conversion rates from December 1, 2022 through December 31, 2024 were calculated by dividing the total number of Veterans called by the total number of Veterans who answered the call and agreed to receive the Boost outreach intervention.

The category of care provided was tallied and the five most common types of care reported.

#### PEEAS

The mean, standard deviation, median and interquartile range (IQR) for each of the 19 questions were calculated. We also calculated the mean and standard deviation of the pooled subscale results for empowerment (questions 1, 2, 3, 4, 5, 11, and 12), engagement (questions 6, 8, 9, 16, and 17), and activation (questions 13, 14, 15, 19, 20, 21) (18).

#### Qualitative interviews

We used a Rapid Qualitative Analysis approach developed for health services research settings, which allows for qualitative results to be analyzed concurrently with data collection to inform the development and testing of interventions and implementation strategies (26, 27). Guided by our semi-structured interview guides, we created summary templates organized by topical area for each of the three key partner groups. A qualitative team member (KZ, CM) listened to each audio-recorded interview and populated a summary template by summarizing key points, including participant quotations illustrating main takeaways. To ensure reliability, a second qualitative researcher reviewed the audio recording and primary analyst's summary for accuracy.

To synthesize data, we used matrix analysis, an approach to displaying data to highlight commonalities and differences and to identify patterns and relationships (26, 28, 29). We created matrices and organized each matrix by key partner group to compare findings across the groups. Qualitative team members met weekly throughout the concurrent data collection and data analysis phases to ensure consensus of the findings, in addition to routinely meeting with the larger study team to discuss preliminary results.

## Results

### Administrative data

Between December 2, 2022 and December 31, 2024, 1,007 unique Veterans received outreach attempts and 518 (51%) were successfully contacted.

Demographic data was collected on Veterans who were called in 2024. The majority of Veterans who received outreach calls (both successful and not successful outreach attempts) identified as white/Caucasian and were between 60 and 69 years of age (Table 1).

**Table 1:** Sociodemographic characteristics for successful outreach calls (*n* = 226), failed outreach calls (*n* = 255), those surveyed (*n* = 58), and those interviewed (*n* = 21).

Sociodemographic characteristics	Successfully contacted from January 1 to December 31, 2024 (*n* = 226)	Not successfully contacted from January 1 to January 31, 2024 (*n* = 255)	Surveyed from June 1, 2023 to November 1, 2024 (*n* = 58)	Interviewed from June 1, 2023 to June 1, 2024 (*n* = 21)
Age				
<30	18 (8%)	20 (8%)	2 (3%)	2 (10%)
30–39	39 (17%)	44 (17%)	7 (12%)	3 (14%)
40–49	35 (15%)	63 (25%)	7 (12%)	2 (10%)
50–59	33 (14%)	40 (16%)	12 (21%)	3 (14%)
60–69	59 (26%)	52 (20%)	15 (26%)	4 (19%)
70–79	35 (15%)	27 (11%)	11 (19%)	6 (29%)
80+	7 (3%)	9 (3%)	4 (7%)	1 (5%)
Race				
White	164 (73%)	166 (65%)	47 (81%)	14 (67%)
Black	15 (7%)	11 (4%)	0 (0%)	0 (0%)
American Indian or Alaska Native	4 (2%)	14 (6%)	0 (0%)	1 (5%)
AAPI	6 (3%)	23 (9%)	1 (2%)	0 (0%)
Unknown	35 (15%)	38 (15%)	10 (17%)	2 (10%)
Multiracial	4 (2%)	5 (2%)	0 (0%)	4 (19%)
Ethnicity				
Not Hispanic/Latino	188 (83%)	203 (80%)	47 (81%)	21 (100%)
Hispanic/Latino	14 (6%)	36 (14%)	6 (10%)	0 (0%)
Unknown	23 (10%)	16 (6%)	5 (9%)	0 (0%)
Multiple	1 (.05%)	0 (0%)	0 (0%)	0 (0%)

The most common types of care provided during outreach calls included one or more new specialty care referrals, primary care follow-up coordination, assistance with coordinating existing specialty care (both within the VHA and through community care), assistance with medication management, and reviewing or placing new laboratory or imaging orders (Table 2).

**Table 2:** Key findings, needs addressed during outreach calls, and illustrative qualitative data.

Key findings	Needs addressed during outreach calls	Illustrative quotes
1. Rural clinic settings exacerbate persistent system-level issues related to accessing VHA care.	28 (9%) received assistance with social/financial issues 45 (15%) received linkage to or direct/real-time gynecological care 28 (8%) received transportation assistance	“[The rural patients we serve] tend to be poorer, tend to have more substance abuse issues, tend to have more housing instability. I have some patients that don’t have running water, don’t have plumbing—a few, but they’re there, just living up in the hills in the woods.” (H006)*
		“Just the simple act of phone outreach or video outreach to some of the female veterans in our community, just because we are so remote and our county is vast … I think, the females, I get the sense that a lot of them probably feel somewhat isolated, you know. We have the VFW, and the American Legion, but those are predominantly male organizations and historically at [our rural outpatient] clinic we haven’t had any groups specifically for female veterans. So I think just continued outreach, so that they have a voice, and their concerns and feelings and needs are being heard, I think that's a start.” (C004)**
		“She [the PCP] said, ‘You know the VA clinic in [major city], why couldn't you just go down there?’ First of all, like we'd have to get a hotel because it's too far away. I have four kids, and I have two kids on the spectrum, I have a rescue dog with three legs and we'd have to take the dog with us. It'd be a whole thing. […] We can't afford that. We can't afford the gas, and the hotel, and the food.” (V007)***.
2. The Boost Program is seen as helping to increase comfort and/or build trust with female Veterans accessing VHA care.	50 (17%) received assistance with linkage to mental health resources 147 (49%) received assistance with primary care coordination or follow-u	“[The woman veteran patient I spoke with] has a dual diagnosis where she's working with substance abuse and military sexual trauma and was very reluctant to come back to VA and in talking to her she felt like the outreach call [from the Boost NP] was what made it safe for her to come back, and she still felt uneasy about it but that she was kind of dipping her toe back in the water of seeing what services could be offered to her.” (H005)*
		“So I have heard from a few female veterans that it's been a positive thing to get that call. They felt validated in having that connection and knowing that the VA is concerned about women-specific issues.” (C004)**
		“I feel like she [Boost NP] actually cares about the patients, so that was refreshing. There's other times I feel like, you kind of just get moved through… I appreciate that she took the time to talk to me and actually listen, like, put in referrals and stuff to get me seen, because I was having trouble just getting anyone on the phone even let alone an appointment. So, she helped a lot.” (V002)***
3. The Boost NP acts as care provider, advocate, and case manager who helps Veterans navigate geographic and institutional barriers to care.	136 (57%) had one or more outstanding health care maintenance item(s) addressed during the outreach call (e.g., toxic exposure screen, cancer screening, osteoporosis screening, depression screening, etc.)	“A lot of patients feel like they’re knocking on the door and calling, and always getting the wrong answer, and here they have a provider calling them and asking if everything's okay and if we can do anything, so I think that proactive approach is making a big difference.” (H010)*
		“In our clinic… we don’t have a dedicated women's health clinic set aside… So I’ve been really supportive of the Boost [program], because the Boost program is able to fill in some of those gaps especially for patient education and patient advocacy, that we may not be able to necessarily meet at our [rural outpatient clinic] for a variety of reasons.” (C005)**
		“I was kind of in this horrible cycle of calling community care, the surgeon was kind of out of the loop at that point, and because the surgery had been done through community care there wasn’t anybody that I could talk to in the orthopedic office that could authorize an extension to the [physical therapy] referral, and the physical therapist was very cooperative and making calls but it was like me calling, wait a couple weeks, me calling, waiting a couple more weeks, me calling, and it was super frustrating… my primary care provider at [my local VA outpatient clinic]—who is great—and her nurse… they were great, but the first time they turned in the paperwork [for the extension] it was disapproved because of something they put in… it was just this cycle of not getting the support… That was the main thing [the Boost NP helped me with] that was super helpful.” (V011)***
4. The Boost NP is seen as added layer of support for under-staffed clinics and as part of the care team.	156 (52%) received one or more new referrals 76 (25%) received assistance with medication management; 48 (16%) received lab/imaging review and/or orders 123 (37%) received coordination with specialty care services at SFVA; 113 (34%) received community care referrals and/or coordination	“I see it as just another layer of support. A provider only is aware of the issues that come right in front of them, like if someone comes in for an appointment or makes a request, then we become aware. It's easy to lose track of what you don’t see, it's easy to not see certain things, so just having that extra layer of support of proactive outreach… is really helpful on the care coordination front.” (H011)*
		“Over the last year we’ve had a lot of staff changes, we’ve had a lot of staff turnover, leadership turnover, there's just been a lot of staffing disruption overall which has been a challenge and so it's nice to have more people on the extended team [like] the Boost team.” (C005)**
		“I’d been wanting to change primary care doctors, and that gave me the opportunity to do that. And [Tara] was very quick with that referral. So yes, my experience [in the VA] is definitely improved… I had been wanting to do it [change providers] for quite some time and hadn't. So it made it easier for her to do it than I think me, you know, going to my local VA and telling them I didn't like one of their doctors.” (V010)***

*Health system leader; **Clinic staff; ***Veteran.

### PEEAS

PEEAS data from 58 Veterans demonstrated strong agreement across all domains which included patient satisfaction, empowerment around diagnoses and treatment, engagement in self-care skills, and activation in self-management (median = 5 or “strongly agree”, IQR = 0 for all 19 questions) (Table 3). We also evaluated means and standard deviations of the pooled subscale responses for the empowerment, engagement, and activation domains (Table 3).

**Table 3:** Patient empowerment, engagement, and activation survey (PEEAS) data (*n* = 58).

Survey question	Mean (standard deviation)	Median	Interquartile range
Empowerment	3.62 (1.87)		
1. I was asked what I already knew about my illness or condition.	2.18 (2.36)	5	0
2. I was told about my medicines before they were prescribed or given to me.	1.88 (2.40)	5	0
3. I was given information or education about my care.	4.31 (1.61)	5	0
4. I was encouraged to ask questions of my health care team.	4.57 (1.27)	5	0
5. I felt I was an important part of the health care team.	4.39 (1.54)	5	0
6. I understand my diagnosis, medical illness, or condition.	3.80 (2.11)	5	0
7. I have a good understanding of my treatments, equipment, or medications to stay well.	4.20 (1.80)	5	0
Engagement	3.79 (1.48)		
8. My doctors, nurses, and other care team members talked with me not about me.	4.18 (1.76)	5	0
9. I took part in my activities and goals.	4.14 (1.84)	5	0
10. I felt comfortable asking my health care team questions about my illness, condition, or care.	4.69 (1.14)	5	0
11. I listened to my healthcare team, they listened to me.	4.53 (1.29)	5	0
12. I was involved in my care or treatment as much as I wanted.	4.51 (1.35)	5	0
13. I took part in decisions about my care or treatment.	4.47 (1.47)	5	0
Activation	3.85 (1.38)		
14. I know when and how to get help or answers to my questions (call the doctor, nurse, hotline, go to ER or urgent care).	4.43 (1.49)	5	0
15. I know what is needed and how to get it for my care at home (equipment, medicines, or help at home).	4.21 (1.71)	5	0
16. I know when my follow-up appointments are.	4.63 (1.23)	5	0
17. I am able to take my own medications.	4.71 (1.19)	5	0
18. I am able to contact my provider (physician, physician assistant, nurse practitioner) if needed.	4.29 (1.66)	5	0
19. I am able to care for myself.	4.80 (0.98)	5	0

PEEAS data demonstrated strong agreement across all categories (median = 5 or “strongly agree”, IQR = 0 for all 19 questions).

### Qualitative interviews

Eleven health system leaders participated in a qualitative interview, including 5 rural CBOC medical directors, 4 health system medical directors, and 5 regional outreach program leads. Five rural outpatient clinic staff members participated in a qualitative interview, including 2 social workers, one primary care provider (PCP), one registered nurse (RN), and one frontline medical support assistant (MSA). Twenty-one Veterans between the ages of 22 and 85 years of age participated in qualitative interviews (Table 1).

Several key findings emerged across 37 key partner interviews: (1) Rural clinic settings exacerbate persistent system-level issues related to accessing VA care (2) The Boost Program is seen as helping to increase comfort and build trust among female Veterans accessing VHA care (3) The Boost NP acts as care provider, advocate, and case manager who helps Veterans navigate geographic and institutional barriers to accessing care (4) The Boost NP is seen as added layer of support for under-staffed clinics and as part of the extended care team (Table 3).

(1) Rural clinic settings exacerbate persistent system-level issues related to accessing VA care

Participants across key partner groups described challenges related to providing and receiving care at VHA CBOCs nested within vast and rural regions. Clinic staff participant C001 explained the impact of rurality in providing care, stating: “It's a very rural area… transportation's an issue, social services are not as prevalent, senior affordable housing is a barrier, memory care, nursing homes. So it's a more challenging demographic [to care for] resource-wise.” Other clinic staff participants highlighted that their outpatient clinics are small, can offer limited services and, in some cases, are chronically understaffed. As one participant noted “We’ve lost two providers, a psychologist, and a psychiatric nurse practitioner just in the last 6 months” (C004). In addition, participants in all key partner groups highlighted that rural VHA CBOCs are limited in specialty care services and thus often require Veterans to travel far distances to the nearest VA medical center located in a major city to access specialty care.

Veteran participants reported several barriers to accessing VHA care, such as long travel times and financial costs associated with traveling to larger VA medical centers, in addition to limited specialty care and women's health care services in their rural communities. In an illustrative example, Veteran participant V007 described how her PCP's initial recommendation to travel to the nearest VA medical center 5 h away for care was unfeasible given the young children and family pets she cares for, all of whom she would need to bring along. Given examples such as these, many Veterans served by rural CBOCs opt for community-based care but often must engage in lengthy referral processes to access these services using their VHA benefits. Though VHA provides referrals to community care services as well as a travel reimbursement program, Veterans described how lengthy referral processes and wait times can result can in Veterans opting to pay out of pocket to access local care more quickly.

Veterans also described challenges related to communication and follow-up when it came to accessing both VHA care and referrals to community-based care. Multiple Veterans shared that when they do not receive follow-up communication about a medical concern after continued attempts, they ultimately stop seeking care for that concern. As one Veteran stated: “No one really did [follow up], I kind of just stopped seeking care for it and just said ‘whatever, it is what it is’” (V006). Given the challenges described by participants in seeking care in rural communities, clinic staff participant C004 emphasized that continued outreach to female Veterans is especially important. In C004's view, not only is the rural community served by their CBOC vast and geographically isolating, many existing veterans' community organizations such as the American Legion and the Veterans of Foreign Wars (VFW) also historically have predominantly male membership which can be further isolating for female Veterans. In this way, C004 stated that the “simple act” of making a Boost program outreach call can help female Veterans have a voice and have their concerns be heard.

(2) The Boost Program is seen as helping to increase comfort and build trust among female Veterans accessing VHA care

Participants across key partner groups expressed concern that female Veterans might feel uncomfortable with, or are resistant to, receiving VHA care. Several Veteran participants echoed this view, noting a perception that the VHA does not focus on offering sex-specific healthcare services (e.g., mammography). Given this, participants across key partner groups reported that receiving an outreach call from the Boost NP can help increase comfort and build trust in VHA care among female Veterans. As one HSL participant explained, “I think the strengths of [the Boost Program] are really creating the milieu by which women can feel safe at VA, because I think it's such a loaded topic getting your care here, especially for Veteran women who may or may not have had good experiences in the military” (H005). In addition, HSL participant H001 highlighted how the Boost Program's telehealth approach can offer female Veterans with histories of military sexual trauma (MST) a pathway to accessing care that makes them feel safer and more secure, stating “I’ve heard that a lot of women feel uncomfortable going into VAs, a lot of them have experienced MST and don’t really want to walk into a [VA] medical center filled with male veterans, so being able to provide that care somewhere where they feel safe and secure is a really great benefit.”

CBOC clinic staff echoed HSL sentiments about the potential impact of the Boost NP's outreach calls. Specifically, clinic staff participant C005 shared her view that the outreach calls help to “bridge the gap in peoples’ knowledge that we do offer women's health and that it's available for Veterans.” Clinic staff participants also noted receiving feedback from female Veteran patients that they experienced the outreach call as positive in that it communicated that the VHA is concerned with sex-specific issues, and that Veterans reported feeling more empowered to access services as a result of receiving the outreach call. Echoing this view, one HSL participant noted that these interactions will support better health for the patient, stating: “Anyone who helps patients feel empowered and engaged is going to be helpful because any patient who is empowered and engaged…is ultimately going to be healthier” (H008).

Veteran participants generally reported that receiving the outreach call had a positive impact or improved their overall experience of VHA care. Several Veterans emphasized the impact of the call in helping them to feel cared about by the VHA as a female Veteran. Veteran participant V002 described receiving the Boost NP's outreach call and care as “refreshing”, in contrast to difficult past experiences attempting to schedule appointments at her local CBOC. For another Veteran who reported that their impression of VHA care was only modestly impacted positively by receiving the outreach call, they shared appreciation for the Boost NP in helping them access additional appointments and for the opportunity to share their feedback with someone at the VHA. One veteran further noted that the Boost outreach call changed a negative impression of VA care they previously held to a positive one, stating: “I felt so much better after hearing from [the Boost NP]” (V0011).

(3) The Boost NP acts as care provider, advocate, and case manager who helps Veterans navigate geographic and institutional barriers to accessing care

Several HSL participants observed that many Veterans find the VA system challenging to navigate, including times when Veterans have an urgent clinical need. HSL participant H010 reflected that with a clinician making the outreach calls, clinical questions can be answered in the moment, medications can be prescribed in the moment, and referrals can be submitted in the moment. Thus, the Boost program model addresses and responds to care needs in real time, a proactive model of care delivery that H010 described as unique in the VHA. H010 added that the receiving an outreach call responded to a common complaint voiced by Veterans–that many experience hurdles to accessing VHA care.

For several Veteran participants, receiving an outreach call from the Boost NP came at a critical time or helped them navigate a tough moment. Veteran V011 described being caught in a frustrating cycle of making phone calls, leaving voice messages, and not getting called back when it came to accessing certain forms of VA-approved community care when the Boost NP intervened. Another Veteran, V003, emphasized that she advocates for herself but encountered many issues attempting to access care at her local CBOC for an urgent matter—so much so that she found herself crying on the phone when Boost NP reached out to her. Following this, the Boost NP made calls on V003's behalf and was able to “get the ball rolling” on accessing the care she otherwise could not access. Across these examples, the Boost NP acted as a care provider, patient advocate, and case manager who helped Veterans navigate both institutional and geographic barriers to accessing care. Given experiences such as these, one clinic staff participant noted the importance of the Boost Program's tailored care approach in addressing each veterans' unique concerns and needs, stating “I think it's personalized, it feels like… Making sure that one specific person, each specific person one at a time, is being taken care of” (C002).

(4) The Boost NP is seen as added layer of support for under-staffed clinics and as part of the extended care team

Overall, HSL and clinic staff shared the view that the Boost NP functions as part of the extended care team and can thus help bridge chronic staffing shortages in the clinic. One clinic staff participant added that “There are some people who live in outlying rural areas that do mainly telehealth, and so particularly in those circumstances where we may not be necessarily seeing someone face-to-face in person once a year, it's good to have as many team members caring for them as we can” (C005). Multiple HSL participants echoed the view that the Boost Program can function as “another layer of support” to primary care teams at the rural VHA CBOCs, with HSL participant H011 elaborating that the Boost program's proactive outreach can help inform primary care teams of patient concerns between appointments as well as help teams with care coordination.

Another HSL participant, H004, similarly noted that the Boost team can act as subject matter experts on specific women's health issues that PCPs might not be experts in, thus providing yet another line of support for providers. Given this, H011 expressed value in having an “outside” person like the Boost NP help keep track of female-specific metrics at her CBOC to help the teams reach their goals in caring for female Veterans. A clinic staff participant, C005, added that the outreach calls not only support clinic staff but similarly bring awareness to Veteran patients by encouraging them to stay current with routine care (e.g., medication refills, routine screenings).

Relatedly, Veteran participants viewed having the Boost NP act as a “3rd party” VA care provider as being helpful or beneficial. Veteran participant V012 offered the example that being more “anonymous” on an outreach call can help someone share openly about concerns related to their care, stating: “Hard to tell a doctor you don’t like the care they’re giving you when they’re your doctor and you don’t have a lot of other choices, you know what I mean?”. Another Veteran participant, V010 shared that the Boost NP reduced barriers to changing her PCP, a process she had been reluctant to initiate on her own. In these ways, the Boost NP's outreach both helps support providers at short-staffed clinics as part of the “extended care team” as well as helps Veterans initiate changes in their care as a “3rd party” care provider.

All participant groups also highlighted how the patient education provided by the Boost NP supports both providers and Veterans. Veterans shared that while the Boost NP can answer specific medical questions, she can also share information about VHA and community resources. As Veteran participant V021 said: “I actually appreciated somebody reaching out and telling me that there were specific services for women veterans.” Clinic staff participant C005 similarly noted that the outreach calls support providers by helping spread the word to patients about new programs. HSL participants echoed this view and emphasized the importance of the patient education because many Veterans “don’t understand the breadth of benefits that they are entitled to” (H008) and the Boost NP's outreach can assist with disseminating this information.

## Discussion

Our findings show that clinician-driven outreach leads to improved patient engagement, empowerment, and activation contributing to restoration of trust in VHA. Quantitative and qualitative data show that Boost succeeds in reconnecting female Veterans back to VHA care, and clinician-driven outreach to Veterans can break down barriers and facilitates improved health. Our mixed-methods programmatic evaluation showed that some of the most valued work done by the Boost NP included motivational interviewing, care coordination, and patient education. Those activities go beyond the scope of simple care navigation and require in-depth medical and system-based practice knowledge. Our findings highlight how, when done well, clinician-driven outreach results in Veterans feeling empowered, engaged, and completing already initiated care plans.

Prior studies show that rural female Veterans face care coordination challenges exacerbated by issues related to community care, a dearth of awareness about VHA sex-sensitive healthcare services, financial barriers, lack of transportation, and difficulties with childcare that all result in decreased utilization of VHA care and drive poor health outcomes (24, 25). Our work demonstrates that clinician-driven telephonic outreach provides vital support resulting in improved VHA experience for rural female Veterans. By leveraging a clinician with women's health expertise, in-depth knowledge of unique and specialized VHA services, and awareness of operational challenges and strengths throughout a health system to provide virtual care across a large geographic range, the Boost NP is able to mitigate many challenges faced by local clinics. As rural clinics continue to suffer from staffing shortages, as gas and travel become more costly, and as many rural community care options close, leveraging highly trained staff based at main VHA medical centers to provide more distributed care may be increasingly necessary to ensure rural Veterans continue to have access to care (30–33). Future studies are needed to identify how best to adapt the Boost Program outreach model to provide care for additional patient populations served by the VHA.

While other studies have used non-clinicians such as Peer Support Specialists (34) to assist in care coordination, what sets Boost apart is that the Boost NP can place orders, answer complex medical questions, and assist in care coordination. The power of this skillset was demonstrated by the Boost NP's impact on gynecologic care. These findings suggest that future work is needed to better identify what subgroups of Veterans most benefit from clinician-driven as opposed to non-clinician-driven outreach.

Our findings also showed how the Boost NP's care coordination helped Veterans advocate and navigate geographic and institutional barriers to accessing care. Because the Boost NP worked at the interface of VHA and community care, we uncovered system-level issues resulting in delays in or lack of care. Prior research shows that women Veterans disproportionately face high rates of delayed or unmet care needs due to burdensome costs, inability to take time off of work, transportation challenges in addition to perceiving VHA care providers to lack women's health expertise and/or sensitivity to military sexual trauma (30). We were able to bring solutions related to care coordination challenges to HSLs, which not only help the individual Veteran being served by Boost, but also improve the system for all end-users. Clinician-driven outreach expands patient-centered care, and we hope to further explore how to scale the Boost Program' clinician-driven outreach model, particularly given the fact that each VA health care system represents a unique ecosystem and solutions that work in one system may not be transferable to others.

Lastly, interviews revealed the view that Boost is seen as acting as an extra layer of clinical support among local CBOC staff. Given the current emphasis on increased throughput and efficiency, Boost may represent a powerful strategy to link at-risk Veterans with unique health needs to specialized clinicians across large geographic areas to provide high-quality care and fill possible knowledge gaps, particularly in rural areas.

This program evaluation provides evidence that clinician-driven outreach can play a powerful role in linking female Veterans to VHA care. Boost works to make the VHA a more welcoming institution for female Veterans by providing individualized and sex-specific care, and clinician-driven outreach is uniquely situated to ameliorate system-level and region-specific barriers to care. As VHA seeks to improve key quality metrics such as completion of routine cancer screening and engage populations of Veterans who traditionally have not sought care in VHA, this evaluation suggests that clinician-driven outreach can be an effective tool to achieve those goals.

This work has both strengths and limitations. This pilot of clinician-driven outreach and quality-improvement focused programmatic evaluation was conducted in one VHA health care system. Although Veterans, HSL, and clinic staff were interviewed across five CBOCs, our findings may not be transferrable to other VHA settings or to non-VA care settings. As such, we are in the process of expanding outreach from the smallest facility in our Veteran Integrated Service Network to the largest. By providing outreach in a new facility across a different geographic region, we aim to better understand what aspects of Boost outreach need to be uniquely tailored to account for regional and health system differences and what aspects are transferable/durable.

## Conclusion

Our findings suggest that clinician-driven outreach positively impacts activation, empowerment, and engagement in VHA care for rural female Veterans and alleviates some of the burden felt by rural clinic staff. In addition, clinician-driven outreach eased health gaps by linking Veterans to specialty services, such as gynecologic care, coordinating care, providing expert-level niche clinical and system-based practice knowledge, and reducing feelings of isolation that can arise from high staff turnover, chronic under-staffing in local clinics, and living in rural regions. Future efforts include expanding geographic range of Boost and better characterizing populations of Veterans that most benefit from clinician-driven outreach.

## Data Availability

The data that support the findings of this study are available from the corresponding author upon reasonable request.
